# Polyelectrolytes Ability in Reducing Atrazine Concentration in Water: Surface Effects

**DOI:** 10.1155/2014/162157

**Published:** 2014-08-14

**Authors:** Mohamad Faiz Mohd Amin, S. G. J. Heijman, S. I. C. Lopes, L. C. Rietveld

**Affiliations:** ^1^Department of Water Management, Faculty of Civil Engineering and Geosciences, Delft University of Technology, 2628 CN Delft, The Netherlands; ^2^Faculty of Earth Science, Universiti Malaysia Kelantan,, UMK Kampus Jeli, 17600 Jeli, Kelantan, Malaysia; ^3^Nalco Europe BV, Ir. G. Tjalmaweg 1, 2342 BV Oegstgeest, The Netherlands

## Abstract

This paper reports on the direct ability of two positively charged organic polyelectrolytes (natural-based and synthetic) to reduce the atrazine concentration in water. The adsorption study was set up using multiple glass vessels with different polymer dosing levels followed by ultrafiltration with a 1 kDa membrane. The addition of polymers exhibited a capability in reducing the atrazine concentration up to a maximum of 60% in surface-to-volume ratio experiments. In the beginning, the theoretical L-type of the isotherm of Giles' classification was expected with an increase in the dosage of the polymer. However, in this study, the conventional type of isotherm was not observed. It was found that the adsorption of the cationic polymer on the negatively charged glass surface was necessary and influential for the removal of atrazine. Surface-to-volume ratio adsorption experiments were performed to elucidate the mechanisms and the polymer configuration. The glass surface area was determined to be a limiting parameter in the adsorption mechanism.

## 1. Introduction 

Micropollutants have become one of the main concerns in environmental pollution because these pollutants are not sufficiently removed in conventional sewage treatment plants. To prevent the spread of such contaminants to surface water and groundwater, the emission of these priority compounds is regulated through the European Water Framework Directive 2000/60/EC [1].

The concern in detecting micropollutants in receiving waters may call for new approaches in wastewater treatment. Wastewater treatment plants are designed to deal with the bulk substances that arrive regularly and in large quantities, which primarily include organic matter as well as nitrogen and phosphorus. Micropollutants are compounds with each having a unique behavior in the treatment plant, and they represent only a minor part of the wastewater organic load [2]. The introduction of a cost-effective method in removing these compounds from wastewater is crucial [3].

The availability of advanced treatment methods has improved the removal of these micropollutants from wastewater, and existing conventional wastewater treatments can be upgraded using such advanced methods [4–6]. However, such treatment methods are often costly. A potential inexpensive solution is the removal of micropollutants in primary sedimentation by coagulation and flocculation [7]. Using polymers as coagulants and flocculants may be advantageous over the use of metal coagulants. A low dosage requirement and a denser sludge production can then lead to cost-effective treatment [8, 9]. The replacement or combination with metal ion coagulants during pretreatment of wastewater can thus be a two-pronged approach: enhancing the micropollutants removal while reducing the costs by 25–30% compared to the use of metal coagulants alone [10, 11].

To develop polymer coagulants, a fundamental understanding of the effect, mechanisms, and ability of polymers to remove micropollutant removal is important. This paper reports on the direct interaction between two polymeric flocculants (synthetic and natural-based) and the micropollutant atrazine (1-chloro-3-ethylamino-5-isopropylamino-2,4,6-triazine) in demineralized water. Adsorption isotherm studies were performed. An L-type of isotherm in the Giles classification was expected with an increase in the dosage of the polymer. However, in dealing with polymers especially polyelectrolytes, this conventional type of isotherm might not be valid because of the dependence on electrostatic and nonelectrostatic interactions [12, 13]. In order to investigate the mechanism surface-to-volume ratio experiments were carried out. Polymer adsorption experiments were also performed to investigate the polymer adsorption pattern on the surface and its effect on atrazine removal. It was hypothesized that the characteristics of the surface play an important role in determining the extent of atrazine removal. In addition, the availability of free opposite charges on the surface of the polymer was expected to influence the adsorption of atrazine.

## 2. Materials and Methods 

### 2.1. Materials

The Nalco cationic polymer 71305 and Nalco cationic starch EX10704 were used and obtained from Nalco Netherlands BV. The Nalco 71305 is a commercially available polymer used as a flocculants aid in wastewater treatment. The Nalco starch is a polymer that is not yet commercially available. The properties of both polymers are listed in Table 1. The polymers were prepared based on standard preparation procedures given by the manufacturer. Atrazine (PESTANAL, analytical standard) and methyl tertiary butyl ether (MTBE, analytical grade) were purchased from Sigma-Aldrich. Demineralized water (DW) was obtained from tap water that was treated by reverse osmosis and ion exchange. The study was performed in DW to exclude other interferences found in (waste) water. A Millipore ultrafiltration membrane (Ultracel regenerated cellulose, 1 kDa NMWL, 47 mm) and a solvent resistant stirred cell (47 mm cell, 75 mL volume) were purchased from Merck Millipore (The Netherlands) for pretreatment of the samples. The membrane was prewashed and stored at 4°C before being used.

### 2.2. Adsorption Studies

Adsorption isotherms were prepared by adding a range of polymer dosages (10, 20, 50, 100, 300, and 500 mg/L of polymer) to 200 mL solutions with concentrations of 23 ± 3 *μ*g/L and 3 ± 0.03 *μ*g/L atrazine in 500 mL Duran glass bottles. Magnetic stirrers were placed inside the bottles, and the bottles were closed. The solutions were stirred at 70 rpm for 24 hours before settling occurred over 3 hours. After the experiment, the sample taken was filtered with the 1 kDa membrane using a solvent resistant stirred cell at air pressure of 5 psi. After each filtration, the membrane was discarded. From a blank experiment, without polymer dosage, it appeared that the effect of the membrane filtration on atrazine concentration could be neglected: the removal was less than 2%. The collected samples after filtration were analyzed for atrazine residues.

### 2.3. Surface-to-Volume Ratio Effect (SVR)

The surface-to-volume ratio (SVR) was studied by dosing 500 ppm Nalco 71305 and starch to 5 *μ*g/L atrazine solutions in 500 mL, 1000 mL, and 2000 mL Duran glass bottles, each with estimated surface area of 353 cm^2^ (SVR 0.7 m^−1^), 547 cm^2^ (SVR 0.5 m^−1^), and 867 cm^2^ (SVR 0.4 m^−1^), respectively. To prove the surface saturation hypothesis, additional experiment was carried out with 1000 ppm Nalco 71305 and starch at SVR values of 1.15 m^−1^ and 1.83 m^−1^. Magnetic stirrers were placed inside the bottles, which were subsequently closed. The solutions were stirred at 70 rpm for 24 hours before settling occurred over 3 hours. After the experiment, the sample taken was filtered using the 1 kDa membrane fitted in solvent resistant stirred cell at air pressure of 5 psi. After each filtration, the membrane was discarded. The collected samples, after filtration, were analyzed for atrazine residues.

### 2.4. Polymer Adsorption

Polymer adsorption was studied to relate the SVR, polymer adsorption, and atrazine reduction. The polymers, Nalco 71305 and starch, each at a concentration of 500 ppm, concentration were added into the Duran glass bottles (filled with demineralized water) with surface areas ranging from 184 to 867 cm^2^. Magnetic stirrers were placed inside the bottles, which were subsequently closed. The solutions were stirred at 70 rpm for 24 hours before settling occurred over 3 hours. Samples were taken and measured using a Shimadzu TOC/V (total organic carbon and total nitrogen analyzer) machine.

### 2.5. Analytical Method

The atrazine concentrations (23 ± 3 and 5 ± 0.2 *μ*g/L) were analyzed by gas chromatography (GC) (Agilent's 7890A) based on the U.S. Environmental Protection Agency 551.1 (1995) method. Atrazine in the sample was extracted using liquid-liquid microextraction with MTBE as a solvent. A total of 1 mL of the extracted sample was used as the injection sample. The carrier gas was helium (the linear velocity was 33 cm/s). The injector temperature was 260°C. The oven temperature was held at 35°C for 9 min and then raised at 15°C/min to 225°C. The temperature of 225°C was held for 10 min before being raised at 20°C/min to 260°C. The GC detection limit for atrazine is 0.01 *μ*g/L. To verify the equilibrium isotherm pattern obtained in the low concentration (3 ± 0.7 *μ*g/L) results acquired by GC, we replicated the experiments and measured the samples using an enzyme-linked immunosorbent assay kit (ELISA) from Abraxis Inc. (USA). The ELISA detection limit for atrazine is 0.04 *μ*g/L. The samples collected after filtrations were injected into the kit based on the recommended procedure of the kit. The kit was then analyzed using a microplate reader (Tecan Infinite 200M Pro). The recovery of atrazine was in the range of 90 to 110% for both methods.

## 3. Results and Discussion 

### 3.1. Adsorption Studies

**Figure 1:**
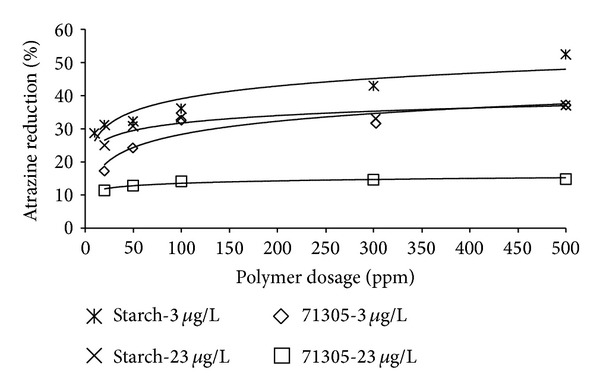
Atrazine reduction percentage of Nalco 71305 and Nalco starch.

**Figure 2:**
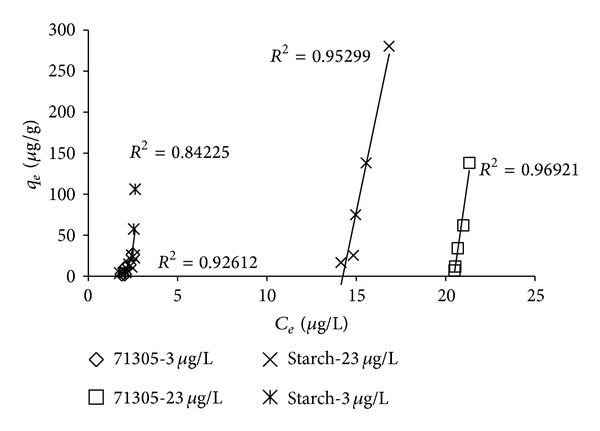
Equilibrium adsorption of atrazine on Nalco 71305 and Nalco starch with different intitial concentrations.

The achieved reduction percentage of Nalco 71305 was in the range of 11 to 15% for the high atrazine concentrations (23 *μ*g/L) and 17 to 37% for the low atrazine concentration (3 *μ*g/L) (Figure 1). The Nalco starch achieved slightly higher reduction ranges of 24 to 36% and 24 to 47% for the high and low atrazine concentrations, respectively (Figure 1). The results also show that the reduction percentage saturates above dosages of 100 ppm for both polymers. There was no clear dose response effect of the different concentrations of polymers (adsorbent) on the removal of atrazine (solute). From the dosed concentration and the removed atrazine, an equilibrium loading *C*_*e*_ (atrazine amount left) was calculated and an adsorption capacity *q*_*e*_ (being the adsorbed amount of the solute per unit weight of adsorbent in *μ*g/g) was determined. The resulting isotherms are shown in Figure 2.

The behavior observed in Figure 2 is not comparable to a conventional isotherm [14]. However, when dealing with polymer adsorption the conventional type of isotherm might not be valid [12, 13]. According to Ghemati and Aliouche [12], a steep increase in adsorption could be caused by a high-energy barrier in the adsorption process that must be overcome before additional adsorption can occur at new sites. However, this adsorption only occurs after saturation of the surface with a monolayer of solutes and is not very likely for adsorption of atrazine to happen onto that surface. The fact that the starting concentration (*C*_0_) influenced the shape of the adsorption isotherm indicates that the explanation should not be found in this direction. The low starting concentration (*C*_0_ is 3.0 ± 0.7 *μ*g/L) resulted in a different isotherm compared to the isotherm measured with a higher starting concentration (*C*_0_ is 23 *μ*g/L). The ELISA and GC methods exhibited comparable results in terms of the pattern of the equilibrium isotherm (results not shown).

### 3.2. Surface-to-Volume Ratio Effect (SVR)

**Figure 3:**
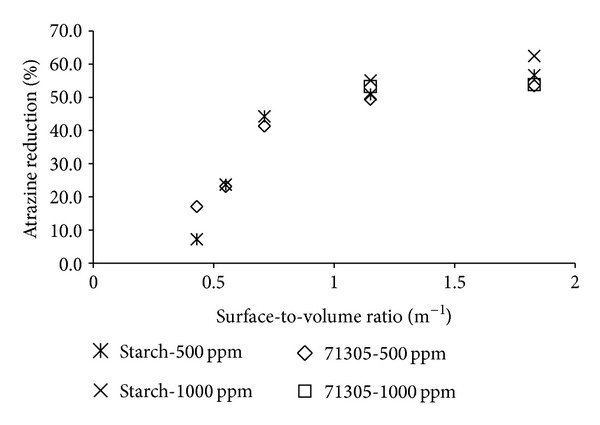
Correlation between the surface-volume ratio and the removal of atrazine by 71305 and cationic starch.

**Figure 4:**
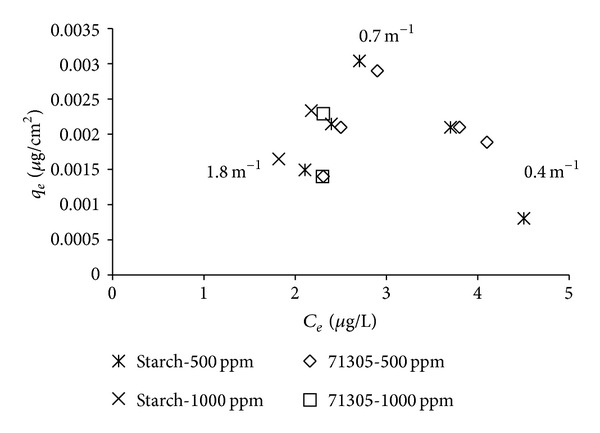
Isotherm of atrazine reduction by 71305 and starch based on SVR (1.8, 0.7, and 0.4 m^−1^).

In accordance with theory and hypotheses already mentioned, we expect that the glass surface area will affect the atrazine reduction (Figures 3 and 4). To prove this effect of the surface-to-volume ratio, an experiment on the effect of the SVR on atrazine reduction was performed.  Figure 3 shows that, with an increase in the SVR, a higher atrazine reduction from the solution occurred before the reduction became constant. With the charge differences between the glass surface (negative) [15] and the polymer (positive), adsorption of polymers to the surface was expected to occur. The adsorption and polymer layer formation on the surface were justified by a lower atrazine concentration reduction with a decrease in the SVR. In relation to the isotherms in Figures 1 and 2, this then resulted in a steep increase in the isotherm and a nearly constant reduction of atrazine. As the SVR increased the glass surface can easily become saturated with polymer molecules. The polymer then has to compete for surface sites. Fleer et al. [16] also reported this phenomenon.

In the context of atrazine reduction, for an SVR of 0.43 m^−1^, the reduction was low due to the spreading and preferable attachment of the polymer to the glass surface. With the increase in the SVR, the polymers had to compete for the surface, thereby leading to polymer multilayer formation, especially for the starch; as a result, the atrazine reduction reached the plateau region, whereby an increase in dosage did not produce noticeable effects. To prove this assumption, the polymer dosages were doubled, to 1000 ppm, in the bottles with SVR values of 1.15 m^−1^ and 1.83 m^−1^ (Figure 3). Beyond the dosage of 500 ppm, we expected to have a limited increase in atrazine reduction. From the data (Figures 3 and 4), the atrazine reduction was observed to increase by only 1 to 7%, which proved the previous surface saturation hypothesis.

From Figure 4, it can be concluded that there was a decrease in atrazine reduction at the surface areas higher than 353 cm^2^ (SVR 0.7 m^−1^). We assumed that the differences in terms of results between starch and 71305 are due to their ionic character.

### 3.3. Polymer Adsorption

**Figure 5:**
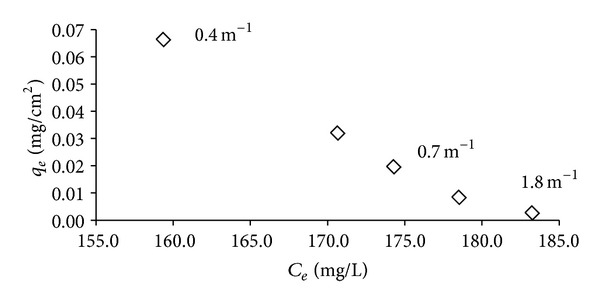
71305 adsorption isotherms based on SVR (1.8, 0.7, and 0.4 m^−1^).

To support the surface saturation theories previously mentioned and the reported theoretical example by Fleer et al. [16], we then measured the polymer adsorption on the surface. In Figure 5, the adsorption amounts of 71305 exhibited a decreasing pattern for the surface areas from 867 cm^2^ to 184 cm^2^, which were in the range of 6.6 × 10^−2^ to 0.3 × 10^−2 ^mg/cm^2^. The achieved 71305 adsorption did not correspond to the atrazine reduction isotherm in Figure 4.

**Figure 6:**
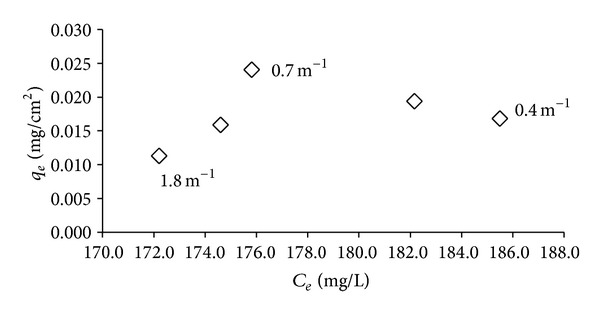
Starch adsorption isotherm based on SVR (1.8, 0.7, and 0.4 m^−1^).

In Figure 6, the starch adsorption was observed to increase at the SVR from 1.8 to 0.7 m^−1^, and then the adsorption was slowly reduced in the range of 0.02 to 0.015 mg/cm^2^ for the SVR values lower than 0.7 m^−1^. The starch adsorption followed the atrazine removal isotherm in Figure 4.

We hypothesized that the behavior of the polymer adsorption and atrazine reduction achieved in this study also depended on the polymer properties, such as electrostatic and nonelectrostatic interactions. Electrostatic interactions may promote or abate polyelectrolyte adsorption onto a charged surface [17]. This interaction is dependent on several interrelated factors, including the surface and polymer charge densities, the salt concentration, and nonelectrostatic interactions, such as Van der Waals and hydrophobic forces [17]. In addition, even with the limitation of the nonelectrostatic interactions, the electrostatic behavior of polyelectrolyte systems is often counterintuitive and cannot be explained with conventional theories of polymers or simple electrolytes [17].

When we compared the polymer adsorption of both 71305 and the starch, we could initially assume that the adsorption of 71305 (Figure 5) was unrelated to the atrazine reduction (Figure 4) compared to the starch adsorption (Figure 6). It was observed that the adsorption of 71305 could be categorized as electrosorption. The adsorption of 71305 did not involve nonelectrostatic interaction, which made it dependent on the surface area and charge [17]. A larger surface-to-volume availability resulted in higher polymer adsorption. Based on this, the higher polymer adsorption should then translate toward higher atrazine removal. However, as shown in Figure 4, the atrazine removal reached a maximum at an SVR of 0.7 m^−1^ and then it decreased for greater SVR values. A similar pattern was also observed in atrazine removal by starch (Figure 4).

For starch adsorption, the interactions involved were both electrostatic and nonelectrostatic. The starch has large-sized molecules that build up to a high-molecular weight polymeric structure. Each of the molecules contains OH-groups that have the ability to react with the surface and the polymer itself. In the case of the low surface areas of 184 to 353 cm^2^ or SVRs between 0.7 and 1.8 m^−1^, the starch was expected to first interact with the surface due to electrostatic interaction. The excess starch then has the ability to attach to the initial adsorbed layer, which is due to nonelectrostatic interactions. This multilayer formation exhibits an energy limit and will deplete at a certain point; it reached a maximum of 353 cm^2^ surfaces area (SVR 0.7 m^−1^) [16].

For both 71305 and starch, when the SVR is lower than 0.7 m^−1^, the larger surface availability resulted in spreading of the polymer onto the surface, whereby the electrostatic effect is dominant. In the 71305 adsorption, the layer was less compact or with a higher spacing between the adsorbed polymers compared to the case of the higher SVR. This higher spacing resulted in a lower atrazine reduction (Figure 4). However, the polymer adsorption was increased (Figure 5), which we expect might be influenced by the 71305 properties. For starch, the lower SVR resulted in less multilayer formation and led toward low polymer adsorption and atrazine reduction. The starch adsorption then further decreased with a larger surface or lower SVR due to the same effect (Figure 6). This decreased starch adsorption then directly affected the atrazine removal whereby the starch adsorption and the atrazine reduction were correlated with each other (Figures 4 and 6).

## 4. Conclusions and Recommendations

The present study was designed to determine the effect and the ability of two different cationic polymers, one synthetic and one natural-based, to reduce the atrazine concentration in demineralized water. Both polymers demonstrated a capacity to reduce atrazine in the water phase, with starch performing slightly better. The isotherm obtained in this study cannot be explained by the typical adsorption behavior of an organic solute on an adsorbent. A possible explanation was that the reduction of atrazine involved two different adsorption mechanisms at the same time: cationic polyelectrolyte attachment to the negative glass surface and atrazine adsorption to polymer layers. In this study, the removal of atrazine was found to be limited by the glass surface availability.

These experiments also led to the following conclusions.

1. The polymers required a negative surface as a support for further adsorption of soluble micropollutants such as atrazine.
2. In practical applications, the polymer can adsorb onto clay particles or other negatively charged particles. These particles can be naturally occurring particles in the wastewater or dosed particles used to increase the amount of micropollutant removal.

## Supplementary Material

The data provided as a support to the figure in the main manuscripts. Focus is given to the main finding that presented in Figure 3-6. Table S1 and S3 is related to Figure 3. Table S2 and S4 is related to Figure 4. Table S5 and S6 is related to Figure 5 and 6 respectively.

## Figures and Tables

**Table 1:** Properties of polymers used in this study.

Product	Description	Form	Solubility (in water)	Ionic character	Molecular weight
Nalco starch EX10704	Modified potato starch	Flaked solid	Soluble	Cationic	10^6^–10^8^
Nalco 71305	Acrylamide copolymer	Emulsion	Soluble	Low cationic	—
