# Supplementary material for: Polyelectrolytes Ability in Reducing Atrazine Concentration in Water: Surface Effects
Source: ScientificWorldJournal. 2014 Aug 14;2014:162157. doi: 10.1155/2014/162157 (PMC4150508; doi:10.1155/2014/162157)
Supplement: Supplementary file 1 — The data provided as a support to the figure in the main manuscripts. Focus is given to the main finding that presented in Figure 3-6. Table S1 and S3 is related to Figure 3. Table S2 and S4 is related to Figure 4. Table S5 and S6 is related to Figure 5 and 6 respectively. [file 162157.f1.pdf]

Supplementary material:

Table S1: Surface to volume ratio during the adsorption experiment by Nalco 71305 (initial 5±0.2 µg/l)

Conc (ppm)	Volume (ml)	Surface area (cm^2)	Surface to volume ratio (m^{-1})	Atrazine final concentration (µg/l) ±0.4	Reduction %
500	100	184	1.83	2.3	53.5
	200	229	1.15	2.5	49.4
	500	353	0.71	2.9	41.3
	1000	547	0.55	3.8	23.1
	2000	867	0.43	4.1	17.1
1000	100	184	1.83	2.3	53.9
	200	229	1.15	2.3	53.5

Table S2: Isotherm of atrazine reduction by Nalco 71305 based on surface area

Conc (ppm)	Area cm^2 (control)	Volume (v) L	C_e µg /L 5±0.2	C0-C = X µg /L	F=X/V µg	F/M = q µg /cm^2
500	184	0.1	2.3	2.6	0.26	0.0014
	229	0.2	2.5	2.4	0.49	0.0021
	353	0.5	2.9	2.0	1.02	0.0029

	547	1	3.8	1.1	1.13	0.0021
	867	2	4.1	0.8	1.66	0.0019
1000	184	0.1	2.3	2.7	0.27	0.0014
	229	0.2	2.3	2.6	0.53	0.0023

Table S3: Surface to volume ratio during the adsorption experiment by cationic starch (initial 5±0.2 µg/l)

Conc (ppm)	Volume (ml)	Surface area (cm^2)	Surface to volume ratio (m^{-1})	Atrazine final concentration (µg/l) ±0.4	Reduction %
500	100	184	1.83	2.1	56.6
	200	229	1.15	2.4	50.9
	500	353	0.71	2.7	44.2
	1000	547	0.55	3.7	23.7
	2000	867	0.43	4.5	7.2
1000	100	184	1.83	1.8	62.5
	200	229	1.15	2.2	55.1

Table S4: Isotherm of atrazine reduction by starch based on surface area

Conc (ppm)	Area cm^2 (control)	Volume (v) L	C µg/L 5±0.2	C0-C = X µg/L	F=X/V µg	F/M = q µg /cm^2
500	184	0.1	2.1	2.7	0.27	0.0015
	229	0.2	2.4	2.5	0.49	0.0022
	353	0.5	2.7	2.1	1.07	0.0030
	547	1	3.7	1.1	1.15	0.0021
	867	2	4.5	0.3	0.70	0.0008

1000	184	0.1	1.8	3.0	0.30	0.0016
	229	0.2	2.2	2.7	0.53	0.0023

Table S5: 71305 adsorption isotherm data

Area<br>cm^2<br>(control)	Volume (v)<br>L	C_e<br>mg/L<br>188.15	C0-C = X<br>mg/L	F=X/V<br>mg	F/M = q<br>mg/cm^2
184	0.1	183.3	4.9	0.5	0.003
229	0.2	178.5	9.7	1.9	0.008
353	0.5	174.3	13.9	6.9	0.020
547	1	170.7	17.5	17.5	0.032
867	2	159.4	28.8	57.6	0.066

Table S6: Starch adsorption isotherm data

Area<br>cm^2<br>(control)	Volume (v)<br>L	C_e<br>mg/L<br>192.85	C0-C = X<br>mg/L	F=X/V<br>mg	F/M = q<br>mg/cm^2
184	0.1	172.2	20.6	2.1	0.011
229	0.2	174.6	18.2	3.6	0.016
353	0.5	175.8	17.0	8.5	0.024
547	1	182.2	10.6	10.6	0.019
867	2	185.5	7.3	14.6	0.017
